# Tubercular Meningitis*Read before the Richmond Academy of Medicine and Surgery, January 26, 1897.

**Published:** 1897-03

**Authors:** J. Allison Hodges

**Affiliations:** Richmond, Va.; Professor of Nervous and Mental Diseases, University College of Medicine, Richmond, Va.


### TUBERCULAR MENINGITIS.*

By J. ALLISON HODGES, M. D., RICHMOND, VA.

Professor of Nervous and Mental Diseases, University College of Medicine, Richmond, Va.

From whatever standpoint viewed, tubercular meningitis is a most interesting disease.

The consideration of its bacillary origin, its peculiar mode of invasion, its stubborn resistance to all known therapeutic measures, its appalling record of mortality, being the most directly fatal of any disease of early childhood, either or all are questions inviting the interest and enthusiasm of every student of disease.

It may almost be called an incurable disease; for, notwithstanding three cases of its happy termination are reported, it

*Read before the Richmond Academy of Medicine and Surgery, January 26, 1897.

is not at all improbable that an incorrect diagnosis was a source of error even with those distinguished clinicians.

Discarding for the present other interesting and instructive features, I desire especially to direct attention to its *diagnosis*, for I consider this of paramount importance, in not so much that its fatal termination can be avoided, as it is at present treated, but that the ends of scientific exactness in this as in other diseases may be attained, and that future errors of prognosis may be avoided.

I am persuaded from personal practice, and from my experience as consultant, that many cases of this disease have been incorrectly interpreted by the attending physician, and the significance of many of its varied symptoms overlooked, until its unfortunate termination revealed the correct nature of the illness. A melancholy experience with two such cases is a lesson still indelibly impressed upon my mind, and it was this that first attracted my attention to my lamentable error in diagnosis; for, deceived by the mild train of symptoms before coma supervened, I overlooked the danger that was impending, and gave an encouraging prognosis! When the symptoms, that shall now be detailed, are considered, is it possible that others may realize that they too may have to plead guilty to such a charge of mistaken diagnosis?

Tubercular meningitis presents a preponderance of nervous symptoms, and is a distinctively bacillary disease, being peculiar to childhood, and usually attacking children between two and ten years of age. The membranes involved are the same as those in simple meningitis, but the anatomical lesions differ, for the latter affects the pia and arachnoid on the convexity of the brain, while a meningitis of tubercular character attacks those membranes at the base.

The prodromal symptoms are vague, and for this reason are apt to be misleading. As much as any clinician, I abhor the division of any disease into arbitrary stages, being well aware how variable are the manifestations, but for the sake of clearness, I prefer to so divide the symptoms of this disease; for, as a rule, there is a regular sequence in them; and it is only from a contemplation of this succession of symptoms, when associated with other manifestations, that the cause of the ominous changes which occur in the later periods is suspected and can be diagnosed.

I believe that too great importance can not be attached to the mode of invasion of this disease as a diagnostic point. In simple meningitis there is a stormy onset, and a convulsion ushers in the scene, while in tubercular meningitis the convulsion is usually one of the last acts in the sad drama. In tubercular meningitis, this period of invasion is so peculiar that it can not be mistaken by the careful observer, if the facts which are of such import in the determination of this particular disease are faithfully elicited.

MODE OF INVASION.

The initiatory symptoms are so indefinite as often to be deceptive. The child is not at once seized with a sudden illness. There is simply a *gradual failing of the general health*, and the only physical sign of disease is a more or less evident emaciation. This change in the condition of the child also is a variable one, being rapid and striking in some, while in others it is scarcely noticeable. In all cases, however, the emaciation at last becomes a marked feature of the case.

During the progress of the change another symptom, which may be termed a *moral change*, develops itself, since it is manifested in both the *habits and temper* of the child; for the little patient, according to its individual temperament, may exhibit all the phases and variations of its nature. Formerly bright and playful, it now becomes morose and sluggish, or irritable and disagreeable. This change of disposition is often supposed to be a reflex condition, due to some primary cause of disease in childhood, such as dentition or some digestive disturbance; but if carefully investigated, no such causative factor is usually discovered.

This moral change in the usual life of the child is not an evanescent one, but is continuously and progressively more marked, and its development should convey a deeper significance to the experienced practitioner.

*First Stage.*—Generally coincident with the moral change, another symptom supervenes which is significant, namely, the existence of a *headache.* This cephalalgia in these cases is characteristic, for it is violent, paroxysmal and generally localized, as evidenced by the actions of the little sufferer. *Vomiting* is usually associated with it, and it is not the ordinary vomiting from gastric disturbances, but the true cerebral vomiting, propulsive or regurgitative in character, unaccompanied by pain or nausea, as, for instance, is the vomiting observed in hepatic or abdominal disorders. These symptoms, accompanied by a mild degree of pyrexia, the temperature most usually exceeding $100\frac{1}{2}°$ F., a slightly accelerated pulse, wakefulness, photophobia, constipation, contraction of the pupils, with possibly some hyperæsthesia of the abdomen, constitute what I would denominate the first stage. In proportion to the intensity of this disease in individual cases will the tardy or rapid succession of the symptoms noted above be manifested, for, generally in affections of the brain, symptoms of irritation and excitation appear first; but sometimes the symptoms of profound depression are initiatory, and this regular succession of phenomena is not so prominent. These prodromal symptoms of the first stage may extend over a period ranging from one to several weeks.

*Second Stage.*—The second stage of the disease (always supposing there is a regular succession of phenomena) ushers in more pronounced symptoms, and there appears to be an increase in their intensity. The temperature rises slightly, and a peculiar *embarrassment of respiration* is noticeable, the patient sighing deeply and long. The pulse now becomes remarkably slow, and there is generally an interference in its rythm which corresponds to the change in the respiration. The eyes half close, and the eyeballs move slowly in a lateral direction, one or both pupils dilating. At this period there are sudden flushes of the face, and a kind of mottling appears upon the skin, over which, if the finger is drawn, red marks are left temporarily; and a sunken, retracted condition of the abdomen is evident. While these two conditions are characteristic in this affection, they are not pathognomonic. During this stage there is also developed a *cephalic cry*, which once heard is never forgotten. The *remission in the fever*, which is apt to occur now, is often deceptive and may lead to false hopes. *Increased somnolence* becomes apparent towards the close of this stage, and in reality is but the beginning of the end.

*Third Stage.*—The foregoing train of symptoms is but introductory to the last stage, where *coma* is the prominent feature, overshadowing all the other conditions. Evidences of vital depression and of organic implication are now more marked, and slight twitchings around the mouth and eyelids, or a convulsion, followed by ptosis, strabismus, or paralysis of the face or extremities promptly follow. The head may become retracted, and all the other symptoms increase in severity till the fatal termination ensues, after a period of three or four weeks.

Not all cases pursue this definite course, for in some children the symptoms may be much milder, while in others there may be so much somnolence from the beginning as to obscure many of them. In fact, to say anything positive about the course of this disease is impossible; it is sometimes acute, sometimes chronic, sometimes presenting long intermissions, and sometimes steadily progressive. Even the manner of onset may vary much, and the different stages or periods so coalesce as to be scarcely distinguishable from each other.

*Differential Diagnosis.*—From the foregoing, it is evident that an absolutely positive diagnosis of tubercular meningitis can be made only when a full and true history of the successive phenomena of the illness can be obtained; or when the symptoms of a tubercular meningitis supervene upon tubercular lesions that have previously existed in other organs. Some of the diseases with which tubercular meningitis may be confounded are:

(1.) Simple meningitis. In this disease, the prodromal symptoms are wanting; the history of tuberculous infection or predisposition is lacking; the onset is more sudden, and the course is more quickly run.

(2.) Cerebro-spinal meningitis. When this disease occurs endomically or sporadically, the vaso-motor disturbances occur earlier, and the characteristic eruption is apt to be present.

(3.) The hydrocephaloid state. This condition in many particulars is so similar to a tubercular meningitis that it can be differentiated only by a history of some gastro-enteric disturbance prior to the supervention of the brain symptoms.

(4.) Malarial fever. The history of malarial infection, or the proper administration of quinine, usually solves the question of diagnosis; otherwise, an examination of the blood must be made for the specific germ.

(5.) Typhoid fever. In this disease, the symptoms of a cerebral character very closely simulate a tubercular meningitis, especially if the temperature curve be atypical, as is frequently the case. A microscopical examination of the blood is often necessary as a crucial test, but, clinically, it is always well to remember that in typhoid there is no peculiar or rythmical disturbance of the respiration, as in meningitis from a tuberculous infection.

*Treatment.*—When the prognosis of this disease can be summed up in one word—death, it is evident that so far no treatment has ever been successful. The multiplicity of remedies suggested corresponds only with their inefficiency.

Recognizing, personally, the utter helplessness of all known measures of therapeusis, as employed in the past, to combat this fatal malady, I purpose in the future to use *anti-tubercle serum*, or if that proves inefficient, to employ *surgical means* to remove the intra-cranial pressure. I am aware that such a procedure would be radicalism in the extreme, but it is a principle which is applied to all other forms of internal pressure and engorgement, and the necessities of such cases demand the application of any measures based on scientific warrant. The ventricles of the brain have been drained and flushed with antiseptic solutions in other diseases, then why not in this? Surely, the phenomenal successes secured in some cases by simply flushing the peritoneum for tubercular disease ought to be encouraging and stimulating to like endeavors in these cases.

In the treatment of this disease, the past is a history of successive and multiplied failures as regard the use of medicines. Is it a vain hope, then, that in the future surgery may solve the mystery of its successful treatment?

